# Hydroxytyrosol Enhances the Nrf2/HO-1 Signalling Pathway to Inhibit Oxidative Stress and Apoptosis and Improve Premature Ovarian Insufficiency In Vitro and In Vivo

**DOI:** 10.3390/ijms27114845

**Published:** 2026-05-27

**Authors:** Shilin Zhang, Yan Xu, Jingxi Zhang, Qingsheng Liang, Zhengdao Chen, Mengyue Zhang, Jingyu Sun, Shaohong Chen, Chuanyin Hu, Yun-Tao Zhao

**Affiliations:** 1 Guangdong Province Engineering Laboratory for Marine Biological Products, Guangdong Provincial Key Laboratory of Aquatic Product Processing and Safety, College of Food Science and Technology, Zhanjiang Municipal Key Laboratory of Marine Drugs and Nutrition for Brain Health, Modern Biochemistry Experimental Center, Guangdong Ocean University, Zhanjiang 524088, China; 17718856452@163.com (S.Z.); 18670244665@163.com (Y.X.); lqs11073731@163.com (Q.L.); 13399181047@163.com (Z.C.); 19000602578@163.com (M.Z.); 15513214868@163.com (J.S.); csh3788@163.com (S.C.); 2 Department of Biology, Guangdong Medical University, Zhanjiang 524023, China

**Keywords:** apoptosis, granulosa cells, hydroxytyrosol, oxidative stress, premature ovarian insufficiency

## Abstract

Premature ovarian insufficiency (POI) poses a serious risk to the reproductive health and psychological well-being of women. Here, the protective effects of hydroxytyrosol (HT), the primary phenolic component of olive oil, on POI were investigated. In vitro, human ovarian granulosa-like tumour cell lines (KGN cells) were challenged by D-galactose (D-gal) with or without HT. HT administration effectively alleviated KGN cell damage, decreased the number of senescence-associated β-galactosidase (SA-β-gal)-positive cells, increased superoxide dismutase (SOD) activity, reduced reactive oxygen species (ROS) and malondialdehyde (MDA) levels, enhanced the expression level of Bcl-2, inhibited the expression level of Bax, and inhibited cell apoptosis in D-gal-treated KGN cells. In vivo, HT administration reversed the decreased ovarian index, oestrous cycle disruption, and abnormal sex hormone levels observed in D-gal-induced POI mice. HT administration increased glutathione (GSH) levels, reduced the MDA levels, and attenuated apoptosis in ovarian tissues, as evidenced by a decreased number of TUNEL-positive cells, upregulated Bcl-2 expression, and downregulated Bax expression. Mechanistically, HT downregulated the expression level of Kelch-like ECH-associated protein 1 (Keap1) and enhanced the expression levels of heme oxygenase-1 (HO-1) and nuclear factor erythroid 2-related factor 2 (Nrf2) in vitro and in vivo. In conclusion, HT ameliorates D-gal-induced POI in vitro and in vivo by activating the Nrf2/HO-1 signalling pathway.

## 1. Introduction

Premature ovarian insufficiency (POI) is defined as a decline in women’s ovarian function, menstrual disorder, or amenorrhea before age 40, accompanied by low estrogen secretion and high levels of gonadotropin [1]. POI can cause a loss of fertility, elevated follicle-stimulating hormone (FSH) levels, and low levels of estrogen in women, which increases the prevalence of osteoporosis, vascular disease, and Alzheimer’s disease. In addition, POI has a detrimental effect on the mental and physical health of women [2]. The incidence rate of POI is around 1–2%, and the prevalence increases each year [3]. The mechanisms underlying the development of POI have not been completely elucidated; research indicates that around 10% of cases have autoimmune, viral, genetic, or iatrogenic causes [4]. Hormone replacement therapy (HRT) is a typical treatment for POI [5]. However, HRT can lead to many serious side effects, including breast cancer, endometrial cancer, vaginal bleeding, abdominal bloating and cardiovascular disease [6]. Thus, it is imperative to elucidate the etiology and pathogenesis of POI and explore effective intervention and treatment methods.

To date, nutritional interventions to improve female reproductive health have been widely recognized [7,8]. Studies have shown that interventions with active ingredients derived from food or Chinese herbs can effectively reduce follicle-stimulating hormone (FSH) levels and increase oestradiol (E2) levels [9,10]. Dietary modification is a simple, economical and healthy intervention approach. The Mediterranean diet (MD) is one of the most respected dietary interventions at present [11].

There is growing evidence that the MD can improve female reproductive health [12,13,14]. The MD is a dietary pattern in which olive oil serves as the primary fat for cooking and seasoning, and there is a high consumption of grains, greens, fruits, and beans, a moderate consumption of seafood, fish, poultry, and nuts, a moderate consumption of milk and dairy products and red wine, and a limited intake of red meat [15]. Olive oil is an important part of the MD and daily consumption of olive oil is beneficial for women’s health, with research indicating that it plays a role in the prevention and treatment of many diseases [16]. In ovariectomized rats, extra virgin olive oil (EVOO) reduces the levels of interleukin-6, malondialdehyde (MDA) and nitrates [17]. EVOO has similar effects to ibuprofen in relieving symptoms of primary dysmenorrhoea, including pain scores and pain duration [18]. Olive oil can also improve sexual intercourse difficulties and alleviate sexual dysfunction in breast cancer patients [19]. However, the specific food ingredients of the MD that can improve female reproductive health remain to be elucidated.

Hydroxytyrosol (HT), the primary phenolic component of olive oil, has various physiological properties including antioxidant, anti-inflammatory, antidiabetic, antitumour, and neuroprotective effects [20,21,22,23,24,25,26]. EVOO consumption, particularly HT, protects the DNA of postmenopausal women from oxidative damage [27]. However, at present, the role of HT in POI is unclear. Here, the impact of HT on the D-galactose-induced POI model, and the underlying mechanisms, were investigated.

## 2. Results

### 2.1. HT Increased the Cell Viabilities and Reduced the Level of Senescence-Associated β-Galactosidase (SA-β-Gal) in D-Galactose-Induced KGN Cells

**Figure 1:**
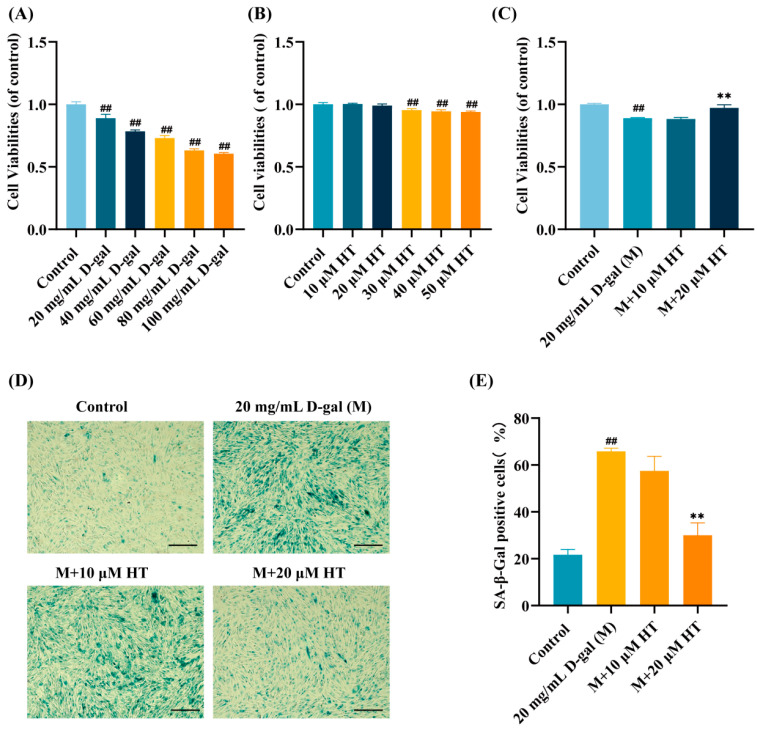
Effects of Hydroxytyrosol (HT) on cell viabilities and the senescence-associated β-galactosidase (SA-β-gal) level of D-gal-challenged human ovarian granulosa-like tumour cell lines (KGN cells). (**A**) Screening of D-gal modelling concentration. (**B**) Screening for non-toxic concentrations of HT. (**C**) Protective effects of HT on KGN cells exposed to D-gal. MTT assays were performed to detect cell viabilities. (**D**) Representative staining diagram of SA-β-gal. The blue cell represents an aged KGN cells. Scale bar: 200 μm. (**E**) Statistical diagram of SA-β-gal-positive cells. The data were presented as Mean ± SEM (*n* = 5). ^##^ *p* < 0.01 vs. control group; ** *p* < 0.01 vs. D-gal group (treated with D-gal).

To determine the toxic impact of D-galactose (D-gal)—a widely used inducer of senescence-like damage—on human ovarian granulosa-like tumour cell lines (KGN cells), cells were challenged with varied concentrations of D-gal (20, 40, 60, 80, and 100 mg/mL) for 24 h, and cell viabilities were measured via the 3-(4,5-dimethylthiazol-2-yl)-2,5-diphenyltetrazolium bromide (MTT) method. The results showed that D-gal markedly reduced cell viabilities of KGN cells in a dose-related manner (Figure 1A). Among the tested concentrations, 20 mg/mL D-gal induced a significant reduction in cell viability while maintaining sufficient cell survival, making it suitable for establishing a stable senescence model without excessive cytotoxicity. Therefore, 20 mg/mL D-gal was selected for subsequent experiments. As seen in Figure 1B, HT at concentrations above 30 μM was toxic to KGN cells (*p* < 0.01). The results revealed that HT intervention effectively inhibited the D-gal-induced cell viability decrease compared to that of the D-gal group (treated with D-gal) (*p* < 0.01, Figure 1C). SA-β-gal is frequently employed to identify senescent cells and serves as a biomarker of the senescent phenotype [28]. Senescent cells were stained blue via SA-β-gal assay (Figure 1D) and the staining images indicated that HT decreased the number of SA-β-gal-positive cells (*p* < 0.01, Figure 1E). These results demonstrate that HT protects KGN cells by restoring cell viability and mitigating cellular senescence, as indicated by the decreased proportion of SA-β-gal-positive cells compared with the D-gal group.

### 2.2. HT Improved Oestrous Cycle Dysfunction and the Reduction in Body Weight in D-Gal-Induced Mice

**Figure 2:**
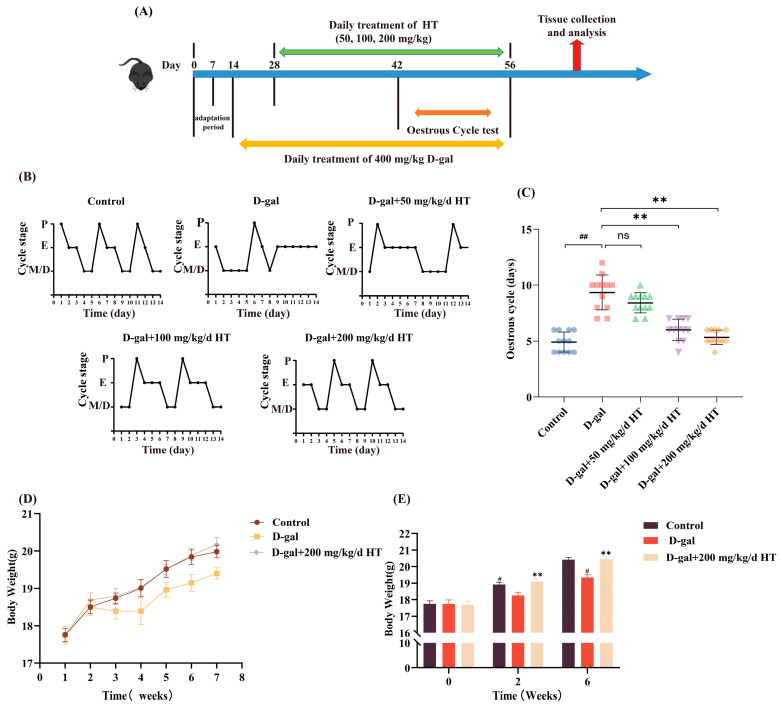
Experimental protocols and impacts of HT on the oestrous cycle and body weight. (**A**) Schematic representation of the animal protocols. (**B**) Typical images of the oestrous cycle in the different groups. The proestrus (P), estrus (E), metestrus (M), and diestrus (D) stages were observed in mice by vaginal smear. (**C)** Length of the oestrous cycle in mice. (**D**) Mouse body weight was assessed during the 6-week period. (**E**) Comparison of body weights at weeks 0, 2 and 6. Data were presented as Mean ± SEM (*n* = 15). ^#^ *p* < 0.05, ^##^ *p* < 0.01 vs. control group; ** *p* < 0.01 vs. D-gal group; ns indicated no significant difference.

To investigate the impact of HT on the oestrous cycle and body weights of D-gal-induced POI mice, mice were administered D-gal (400 mg/kg/day) via subcutaneous injection for 42 days and treated with HT (or vehicle) by intragastric gavage for 28 days starting from the third week (Figure 2A). POI is characterized by disruption of the oestrous cycle [29]. In normal mice, the estrous cycle was generally 4–5 days. Following the D-gal injection, the length of the oestrous cycle was disrupted and prolonged, and the oestrous cycle stages were disordered (*p* < 0.01, Figure 2B,C). In contrast to the D-gal group, mice administered 100 and 200 mg/kg/d HT showed restoration of the oestrous cycle stage and a significant decrease in the length of oestrous cycle (*p* < 0.01, Figure 2B,C). Thus, the results indicate that HT remarkably improved the irregularity of the oestrous cycle in POI mice. As seen in Figure 2D, mice in the D-gal group had different degrees of weight loss starting from the second week, in contrast to the control group. Following the 4-week HT intervention, mice in the 200 mg/kg/d HT-treated group exhibited a notable increase in weight compared to the D-gal group (*p* < 0.01, Figure 2E). These findings suggested that HT exerts a protective effect by normalizing the oestrous cycle and promoting weight recovery in POI mice.

### 2.3. Effects of HT on Sex Hormone Levels, the Ovarian Index, and Ovarian Morphology

**Figure 3:**
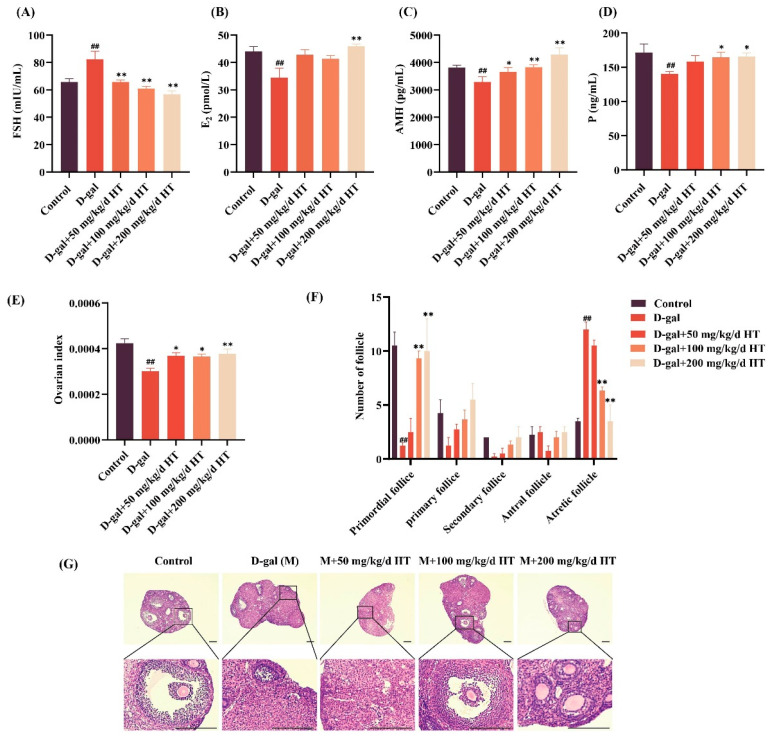
Effects of HT on sex hormone levels, the ovarian index, and ovarian morphology. The concentrations of FSH (**A**), E2 (**B**), AMH (**C**) and P (**D**) were measured via ELISAs. Data were presented as Mean ± SEM (*n* = 5). (**E**) Comparison of the ovarian index in the different groups of mice. The ovarian index is equal to the weight of the ovary divided by the mouse weight. Data were displayed as Mean ± SEM (*n* = 15). (**F**) The number of follicles was recorded and displayed. Data were presented as Mean ± SEM *(n* = 15). (**G**) Representative images of ovarian morphology in the various groups. Scale bar: 200 μm. ^##^ *p* < 0.01 vs. control group; * *p* < 0.05,** *p* < 0.01 vs. D-gal group (M).

The sex hormone levels of the mice were further evaluated to investigate the role of HT in D-gal-induced POI. The ELISA data showed that the FSH level was significantly increased (*p* < 0.01, Figure 3A), and the E2, anti-Müllerian hormone (AMH), and progesterone (P) levels were decreased (*p* < 0.01, Figure 3B–D) in the D-gal group compared with that of the control group. After the HT intervention, the FSH level was considerably reduced (*p* < 0.01, Figure 3A) and the E2 level was greatly increased in the 200 mg/kg/d HT-treated group (*p* < 0.01, Figure 3B) compared with that of the D-gal group. There were no substantial variations in the E2 levels between the 50 and 100 mg/kg/d HT-treated groups. HT administration also significantly increased the AMH and P levels (*p* < 0.05, Figure 3C,D). These data demonstrate that HT effectively reversed the sex hormone level changes in D-gal-induced mice.

The impact of the HT intervention on follicular development in POI mice was evaluated by hematoxylin and eosin (H&E) assays. After D-gal injection, the mice showed a drastically lower ovarian index, fewer primordial follicles, and more atretic follicles (*p* < 0.01, Figure 3E–G) compared with that of the control group. After the HT intervention, the mice exhibited a notable elevation in the ovarian index, a considerable rise in primordial follicles, and a large reduction in atretic follicles (*p* < 0.01, Figure 3E–G). These findings indicate that HT ameliorated the reduction in the ovarian index and improved the abnormalities in ovarian morphology in D-gal-induced mice. These results demonstrated that HT alleviates D-gal-induced POI by normalizing hormonal profiles and preserving ovarian microarchitecture.

### 2.4. HT Reduced D-Gal-Induced KGN Cells Apoptosis

**Figure 4:**
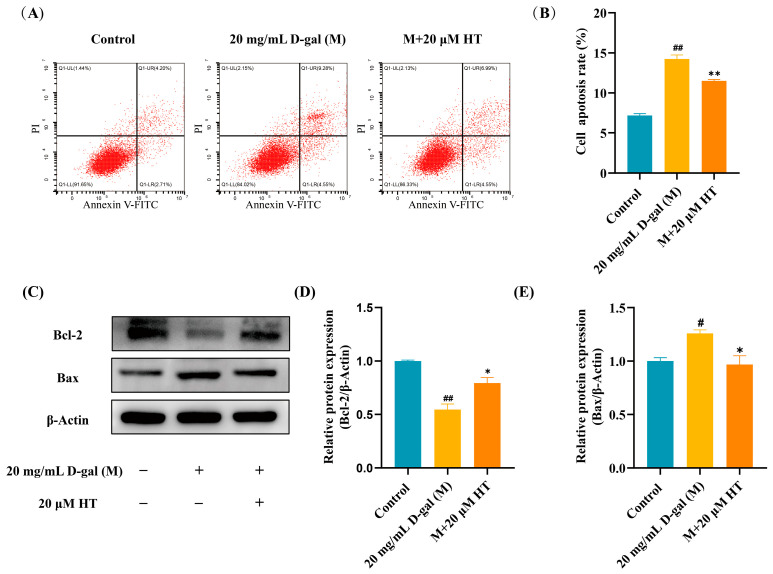
Effects of HT on apoptosis in D-gal-challenged KGN cells. (**A**) Flow cytometry images. (**B**) The cell apoptosis rate was determined. (**C**) Western blot images of the Bax and Bcl-2 proteins. (**D**) Quantitative assessment of the expression level of the Bcl-2 protein. (**E**) Quantitative assessment of the expression level of the Bax protein. Data were presented as Mean ± SEM (*n* = 3). ^#^ *p* < 0.05, ^##^ *p* < 0.01 vs. control group; * *p* < 0.05, ** *p* < 0.01 vs. D-gal group (M).

The anti-apoptotic efficacy of HT was measured in D-gal-challenged KGN cells using flow cytometry and Western blot (Figure 4A,C). The percentage of apoptotic cells in the D-gal group increased to 14% compared with that of the control group, as shown in the flow cytometry images (*p* < 0.01, Figure 4B). HT administration at a concentration of 20 µM resulted in a statistically significant decrease in the extent of apoptosis in KGN cells subjected to D-gal treatment (*p* < 0.01, Figure 4B). Western blot data showed that the expression level of B-cell lymphoma 2 (Bcl-2) was statistically reduced (*p* < 0.01, Figure 4D) and the expression level of Bcl-2-associated X protein (Bax) was statistically upregulated (*p* < 0.05, Figure 4E) in the D-gal group compared to that of the control group. After the HT treatment, the expression level of Bcl-2 showed a statistically significant rise (*p* < 0.01, Figure 4D), whereas the expression level of Bax showed a substantial decrease (*p* < 0.05, Figure 4E), in comparison to the D-gal group. These results indicated that HT protects KGN cells from D-gal-induced injury by inhibiting programmed cell death.

### 2.5. HT Inhibited the Apoptosis of Mouse Ovarian Granulosa Cells (GCs) Induced by D-Gal

**Figure 5:**
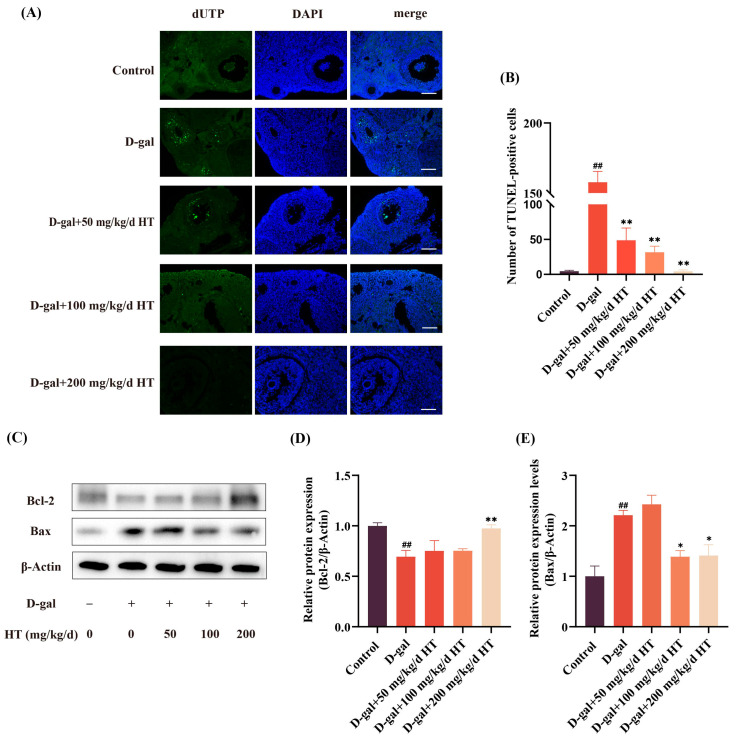
Effects of HT on apoptosis in D-gal-induced mice. (**A**) Ovarian TUNEL fluorescence images, scale bar: 100 μm. (**B**) Graph of apoptosis in ovarian granulosa cells. (**C**) Western blot images of Bax and Bcl-2. (**D**,**E**) Quantitative assessment of the expression levels of Bax and Bcl-2 in the ovaries. Data were presented as Mean ± SEM (*n* = 3). ^##^ *p* < 0.01 vs. control group; * *p* < 0.05, ** *p* < 0.01 vs. D-gal group.

In order to examine ovarian GC apoptosis, green fluorescence was applied to the nuclei of TUNEL-positive cells in ovarian paraffin sections (Figure 5A). The effects of HT on the expression levels of Bax and Bcl-2 in the ovarian tissues were detected via Western blot (Figure 5C). Compared to the control group, the quantity of TUNEL-labelled positive cells in the D-gal group was significantly increased (*p* < 0.01, Figure 5B). The TUNEL-labelled positive cell count was considerably reduced in the HT-treated groups (*p* < 0.01, Figure 5B). These results revealed that HT inhibited the apoptosis of ovarian GCs induced by D-gal. The expression level of Bax was statistically increased (*p* < 0.01, Figure 5C,D) and the expression level of Bcl-2 was statistically reduced (*p* < 0.01, Figure 5C,E) in the D-gal group compared with that of the control group. HT administration rescued the D-gal-induced apoptosis of ovarian tissues in POI mice (*p* < 0.05, *p* < 0.01, Figure 5D,E). These in vivo data demonstrated that HT preserved ovarian function by mitigating granulosa cell apoptosis and modulating apoptosis-associated protein expression.

### 2.6. HT Alleviated Oxidative Stress in D-Gal-Induced KGN Cells and Mice

**Figure 6:**
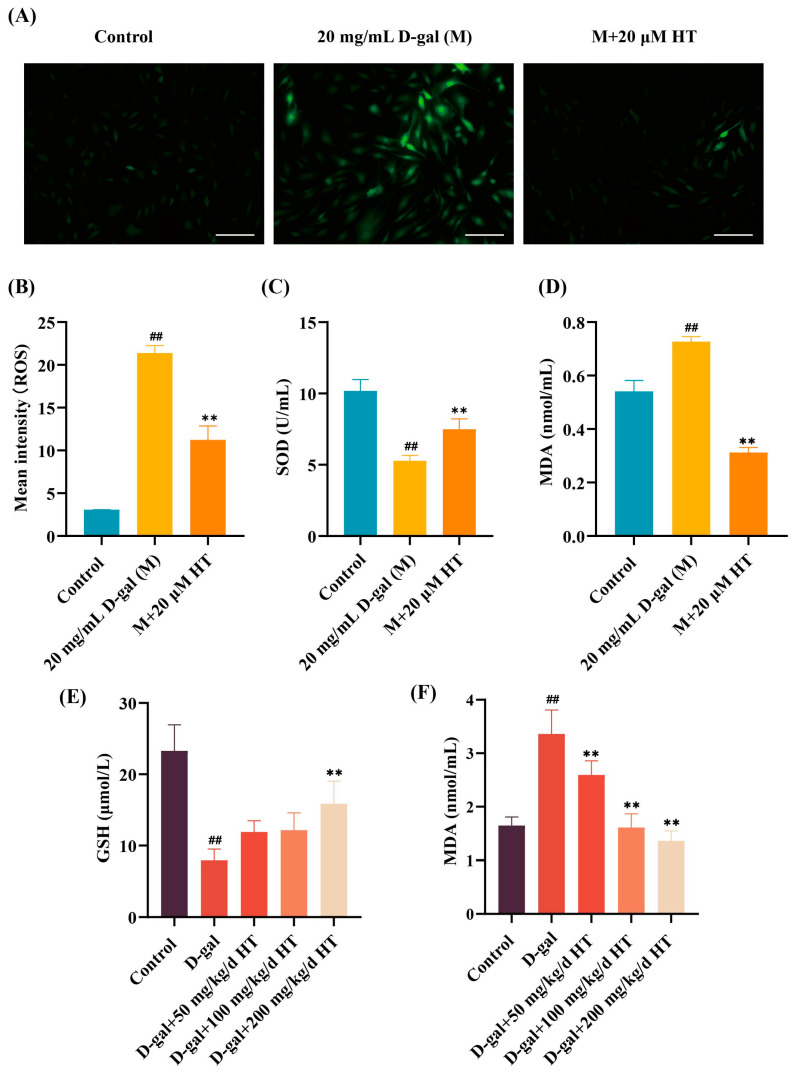
Effects of HT on oxidative stress in D-gal-challenged KGN cells and mice. (**A**) ROS fluorescence images. ROS production was measured and analyzed using the DCFH-DA probe. Scale bar: 100 μm. (**B**) Immunofluorescence histogram expressing the mean intensity of ROS. (**C**) The SOD contents (U/mL) and (**D**) MDA levels (nmol/mL) were measured in KGN cells. (**E**) The GSH (μmol/L) activities and (**F**) the MDA (nmol/L) levels were quantified in the ovarian tissues of the various groups. The data were displayed as Mean ± SEM (*n* = 3). ^##^ *p* < 0.01 vs. control group; ** *p* < 0.01 vs. D-gal group (M).

Oxidative stress plays a role in the occurrence and development of POI. Reactive oxygen species (ROS) accumulation is an important indicator of oxidative stress. In vitro, as seen in Figure 6A, D-gal treatment caused a notable increase in the ROS levels (*p* < 0.01, Figure 6B). The HT intervention successfully decreased the ROS level (*p* < 0.01, Figure 6B). In D-gal-induced KGN cells, superoxide dismutase (SOD) activity was greatly decreased (*p* < 0.01, Figure 6C), whereas the MDA level was substantially increased (*p* < 0.01, Figure 6D), in comparison to the control group. Following the HT intervention, there was a significant elevation in SOD activity (*p* < 0.01, Figure 6C) and a notable drop in MDA levels (*p* < 0.01, Figure 6D). In vivo, glutathione (GSH) level was significantly reduced (*p* < 0.01, Figure 6E) and the MDA level was significantly increased (*p* < 0.01, Figure 6F) in the D-gal group compared with that of the control group. Moreover, antioxidant GSH activity was considerably improved in the 200 mg/kg HT-treated group (*p* < 0.01, Figure 6E) and the MDA levels were markedly reduced in HT-treated groups (*p* < 0.01, Figure 6F). In summary, HT administration restored the equilibrium of redox reactions in vitro and in vivo when challenged by D-gal.

### 2.7. HT Alleviated D-Gal-Induced POI via the Nrf2/HO-1 Signalling Pathway In Vitro and In Vivo

**Figure 7:**
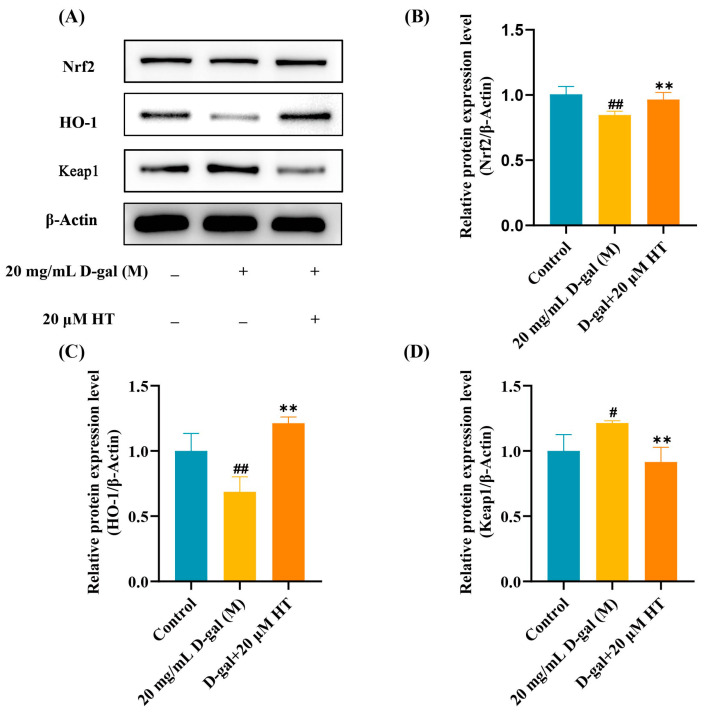
Effects of HT on the Nrf2/HO-1 signalling pathway in D-gal-treated KGN cells. (**A**) Western blotting result of the Nrf2/HO-1 signalling pathway. (**B**) Quantitative analysis of Nrf2 protein expression. (**C**) Quantitative analysis of HO-1 protein expression. (**D**) Quantitative analysis of Keap1 protein expression. ^#^ *p* < 0.05, ^##^ *p* < 0.01 vs. control group; ** *p* < 0.01 vs. D-gal group.

**Figure 8:**
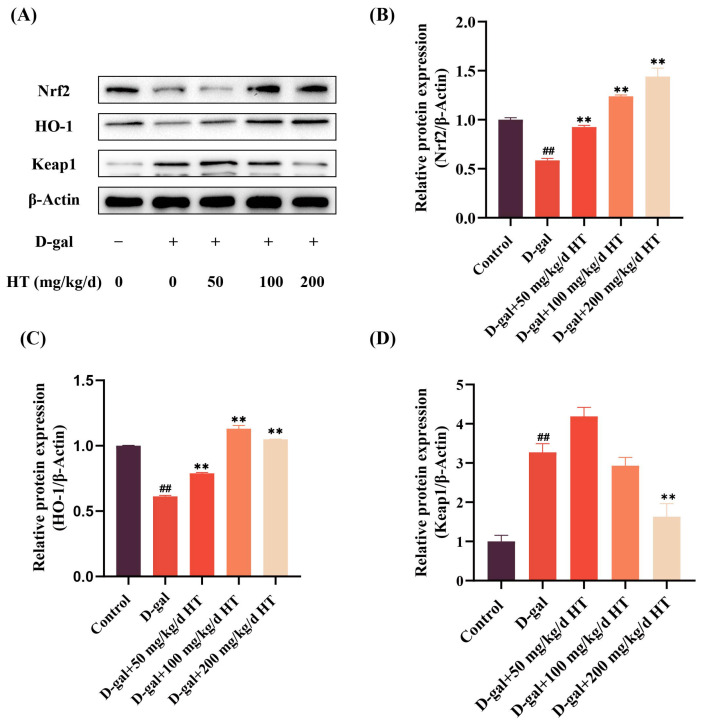
Effects of HT on the Nrf2/HO-1 signalling pathway in D-gal-induced mice. (**A**) Western blot images of the Nrf2, HO-1 and Keap1 proteins. (**B**–**D**) Quantitative assessment of the Nrf2, HO-1 and Keap1 expression levels in the ovaries. Data were shown as Mean ± SEM (*n* = 3). ^##^ *p* < 0.01 vs. control group; ** *p* < 0.01 vs. D-gal group.

The Nrf2/HO-1 signalling pathway is a significant internal antioxidant system [30]. Nuclear factor erythroid 2-related factor 2 (Nrf2) is a key transcription factor that regulates oxidative stress in the ovaries and is an essential transcription factor in the regulation of cellular oxidative stress [31]. Activation of Nrf2 can protect cells from damage caused by ROS and stabilizes cells by regulating the downstream expression of the antioxidant enzyme heme of heme oxygenase-1(HO-1) [32]. Western blot analysis was used to assess the expression levels of Nrf2, HO-1, and Kelch-like ECH-associated protein 1 (Keap1) in D-gal-induced POI in vitro and in vivo (Figure 7A and Figure 8A). In vitro, the expression levels of Nrf2 and HO-1 were considerably decreased (*p* < 0.01, *p* < 0.01, Figure 7B,C), whereas the expression level of Kelch-like ECH-associated protein 1 (Keap1) was remarkably elevated in the D-gal group compared to that of the control group in vitro (*p* < 0.05, Figure 7D). The HT intervention significantly reversed these alterations in vitro (*p* < 0.01, Figure 7B–D).

In vivo, the expression levels of Nrf2 and HO-1 were considerably decreased (*p* < 0.01, *p* < 0.01, Figure 8B,C), whereas the expression level of Keap1 was remarkably elevated in the D-gal group compared to that of the control group (*p* < 0.01, Figure 8D). The HT intervention significantly reversed these alterations in D-gal-induced POI mice (*p* < 0.01, Figure 8B–D). These results demonstrated that HT exerted its protective effects against D-gal-induced oxidative stress by activating the Nrf2/HO-1 signalling pathway both in vitro and in vivo.

## 3. Discussion

POI exerts a considerable physical and mental burden on women. Previous research has demonstrated that dietary interventions have a protective impact on POI. HT, a major phenolic compound derived from olive oil, is of benefit to women’s health [33,34]. Here, we aimed to explore the potential effects of HT on a POI model, and the underlying mechanisms, in vitro and in vivo.

D-gal is extensively utilized by researchers in both in vivo and in vitro aging models, including those involving the brain, liver, kidneys, and ovaries [35,36,37]. Here, D-gal treatment increased the quantity of SA-β-gal-positive cells and reduced the viability of KGN cells. The HT intervention significantly inhibited the D-gal-induced decrease in the viability of KGN cells and reduced the number of SA-β-gal-positive cells, suggesting that HT has an anti-aging effect and protects the cells from the cellular damage caused by D-gal. Subcutaneous injection of D-gal is a typical senescence model in mice. D-gal is converted into galactitol through the action of aldose reductase. However, galactitol cannot be further metabolized. The accumulation of galactitol in cells causes cell swelling and metabolic problems, and ultimately triggers cellular senescence [5]. D-gal is a major cause of primary or secondary amenorrhoea in women, and an increase in D-gal leads to a decrease in ovarian follicles, damage to oocytes and GCs, and a decline in ovarian function [38]. Based on previous studies, 400 mg/kg/d D-gal was used in this study to establish a POI mouse model [39]. The results showed that subcutaneous injection of D-gal induced a decrease in the ovarian index and disruption to the oestrous cycle of mice. The HT intervention significantly increased the ovarian index and restored the oestrous cycle in D-gal-induced POI mice.

The oestrous cycle is observed in the majority of female animals. It involves regular physiological changes driven by hormonal fluctuations and plays an important role in female development [40,41]. Disordered oestrous cycles can cause hormonal disorders, menstrual irregularities, and amenorrhea. Oestrous cycle disorder is an important feature in POI. In normal mice, the oestrous cycle usually lasts for 4–5 days. In our study, D-gal-treated POI mice exhibited disrupted oestrous cycle regularity, with prolonged or irregular cycle lengths, consistent with the hormonal dysregulation characteristic of POI. Notably, HT intervention restored the regularity and length of the oestrous cycle in POI mice, suggesting a recovery of hypothalamic–pituitary–ovarian (HPO) axis function. The incidence of POI is strongly correlated with the levels of secreted sex hormones produced by the hypothalamic–pituitary–gonad (HPG) axis. The HPO axis regulates female reproductive function and balance. FSH is required for follicular maturation and stimulation of ovarian estrogen production, and is also necessary for the production of P and E2 [42]. AMH is a key biomarker of ovarian reserve. POI is defined by low levels of E2, increased levels of FSH > 25 IU/L, and amenorrhoea or oligomenorrhea lasting at least 4 months [42]. Hence, it is important to monitor blood levels of FSH, E2, AMH and P in cases of POI. In the present study, D-gal-treated POI mice showed elevated FSH and reduced E2, AMH, and P levels, consistent with impaired ovarian reserve and folliculogenesis. Elevated FSH reflects a compensatory pituitary response to diminished ovarian feedback, while reduced AMH and E2 indicate follicular depletion and insufficient steroidogenic activity [43,44]. These hormonal changes were corroborated by a decreased ovarian index, reduced follicle counts, and disrupted oestrous cycles. Following HT intervention, FSH levels declined and E2, AMH, and P levels were restored, accompanied by a recovery of follicle numbers and oestrous cycle regularity, suggesting that HT restores HPO axis function and ameliorates D-gal-induced ovarian dysfunction by preserving ovarian reserve and promoting follicular development.

Studies have shown that oxidative damage can cause ovarian dysfunction [45]. As individuals age, there is increased production of harmful free radicals and a decline in the presence of antioxidants in the ovaries, which can hinder the ability of the ovaries to eliminate these free radicals [39]. D-gal, as a reducing sugar, readily interacts with the free amines of amino acids in proteins and peptides to form advanced glycation end-products (AGEs) in vivo and in vitro [46]. Excess D-gal induces oxidative stress, ROS accumulation, lipid peroxidation, and glucose oxidation, which promotes the production of AGEs. An increase in AGEs accelerates the aging process [47]. A growing body of evidence suggests that oxidative stress is a crucial factor in the pathophysiology of POI [48,49,50]. During oxidative stress, the antioxidant defence mechanisms of organisms are frequently triggered to guard against oxidative damage by preserving cellular redox equilibrium [51,52]. ROS accumulation leads to a reduction in the antioxidant capacity, which causes ovarian and severe cellular damage [53]. SOD plays a crucial function in mitigating oxidative stress by eliminating excessive ROS, thereby safeguarding cells against oxidative damage. GSH is the most important non-enzymatic antioxidant in organisms and is an important substrate for the decomposition of hydroperoxides by GSH-PX and GSH-ST, scavenging O_2_^−^, H_2_O_2_, and LOOH [54,55]. MDA is a lipid peroxide that is formed when oxygen radicals attack polyunsaturated acids in biological membranes [56]. In our investigation, D-gal stimulation increased the ROS level, increased the MDA level, and decreased the SOD activity of KGN cells. D-gal significantly reduced the antioxidant capacity of the ovaries, which was characterized by a considerable increase in the MDA content and a decrease in the GSH activity of ovarian tissue, usually considered an indicator of the oxidative stress response. HT administration significantly attenuated these changes in oxidative damage parameters in vitro and in vivo.

ROS accumulation is an important indicator of oxidative stress which triggers apoptosis [57]. Apoptosis of ovarian GCs is the main mechanism underlying impaired ovarian function [58,59,60]. GCs generate a range of substances that support oocyte development and maturation [61]. Furthermore, oocytes release cytokines that interact with the appropriate receptors on the surface of GCs, thereby promoting GCs proliferation and differentiation [62]. Apoptosis of GCs induces damage to oocytes, leading to accelerated follicular atresia and POI [63]. To investigate the apoptosis of ovarian GCs in mice, in situ TUNEL analysis was performed to stain the nuclei of TUNEL-labelled positive cells in mature follicles. The experimental results showed that HT alleviated POI-associated abnormalities in the D-gal-induced mouse model by attenuating granulosa cell apoptosis. In vitro, D-gal-induced cell apoptosis in KGN cells. After HT treatment, the ratio of early to late apoptosis was significantly reduced. Bcl-2 is an anti-apoptotic protein that plays an important role in stabilizing the mitochondrial membrane and Bax oligomerizes and accelerates the release of cytochrome C into the cytoplasm under stressful conditions [64,65]. There is a clear relationship between the Bcl-2/Bax ratio and ovarian granulocyte apoptosis [66]. Additionally, research has shown that the inactivation of Bax can prevent ovarian damage caused by smoking [67]. The current study revealed a decline in the expression of the Bcl-2 protein and an elevation in the expression levels of the Bax protein in vitro and in vivo when exposed to D-gal. HT inhibited the D-gal-induced apoptosis of GCs by increasing Bcl-2 expression and suppressing Bax hyperexpression.

The Nrf2/HO-1 pathway has a significant effect on the expression of genes involved in antioxidant and anti-apoptotic functions [32,68,69]. Nrf2, an intranuclear transcription factor, promotes the expression of downstream antioxidant proteins, such as HO-1 and SOD, after entering the nucleus, whereas Keap1 negatively regulates Nrf2 through ubiquitination and degradation, preventing Nrf2 from entering the nucleus [70,71]. Lim et al. demonstrated that Kaempferol-3-O-β-d-Glucuronate effectively inhibits ROS production via its ability to increase Nrf2/HO-1 signalling [72]. The Nrf2/HO-1 pathway is also thought to contribute to apoptosis regulation [73]. In this study, it was revealed that the Nrf2/HO-1 signalling pathway was suppressed and the antioxidant capacity was compromised in cells and mice. Following the HT intervention, there was a notable rise in the expression levels of Nrf2 and HO-1, and the expression level of Keap1 was significantly decreased. These results demonstrated that HT effectively increased the antioxidant capacity of D-gal-induced POI models both in vitro and in vivo by activating the Nrf2/HO-1 signalling pathway. Nevertheless, although reciprocal modulation of Keap1 and HO-1 levels supports activation of the Nrf2/HO-1 axis, the lack of Nrf2 nuclear translocation evidence and loss-of-function or rescue experiments limits confirmation of its causal role in HT-mediated protection.

It should be noted that HT exerted cytoprotective effects within a defined concentration range; concentrations exceeding 30 μM were cytotoxic to KGN cells, highlighting the importance of appropriate dosing. The concentration selected in this study (20 μM) remains below the cytotoxic threshold and falls within physiologically relevant plasma levels reported after oral HT intake, supporting its biological relevance. However, following oral administration, HT is rapidly absorbed and extensively metabolized, leading to low systemic exposure to its free form, which may limit its in vivo efficacy. Delivery systems such as organogel emulsions and solid lipid nanoparticles have been shown to improve its bioavailability and enhance biological activity [74,75]. Additionally, the present study provides associative rather than causal evidence for the Nrf2/HO-1 axis in HT-mediated protection, as loss-of-function or rescue experiments were not performed [39]. Furthermore, KGN cells, as a human granulosa-like tumour cell line, may not fully recapitulate normal granulosa cell physiology, and future studies using primary granulosa cells are warranted to further validate these findings. Therefore, subsequent investigations should focus on developing optimized delivery strategies for HT to improve its bioavailability, thereby further validating the protective effects observed in our experimental models and providing a stronger preclinical basis for future research on POI.

## 4. Materials and Methods

### 4.1. Reagents

D-(+)-galactose (G0750) was obtained from Sigma-Aldrich (St Louis, MO, USA). HT (S25176) and 3-(4,5-dimethylthiazol-2-yl)-2,5-diphenyltetrazolium bromide (MTT, S19063) were purchased from Shanghai YuanYe Biotechnology Co., Ltd. (Shanghai, China). 2′, 7′-Dichlorodihydrofluorescein diacetate (DCFH-DA, C2938) was obtained from Invitrogen (Carlsbad, CA, USA). Fetal bovine serum (FBS) and DMEM/F-12 basic medium were supplied by Gram Island Biological Company (Carlsbad, CA, USA). BCA protein (P10012), senescence-associated β-galactosidase (SA-β-gal, C0602), and Annexin V-FITC Apoptosis (C1062L) assay kits were bought from Beyotime Technology Co., Ltd. (Shanghai, China). Mouse FSH (JM-02838M2), E2 (JM-02849M2), AMH (JM-11692M2) and P (JM-02851M2) ELISA kits were acquired from Jiangsu Jingmei Biological Technology Co., Ltd. (Yancheng, China). A SOD kit was got from Suzhou Grace Biotechnology Co., Ltd. (Suzhou, China). MDA and GSH kits were available at Jiancheng Bioengineering Institute (Nanjing, China). A One Step TUNEL In Situ Apoptosis Detection kit was supplied by Elabscience Biotechnology Co., Ltd. (Wuhan, China). Bcl-2 (SC-7382) and Bax (SC-7480) antibodies were obtained from Santa Cruz (Dallas, TX, USA). Abcam (Cambridge, UK) supplied the antibodies for Nrf2 (AB96946) and HO-1 (AB13243) antibodies. Boster Biological Technology Co., Ltd. (Wuhan, China) provided the Keap1 (A00514-3) antibody. The β-Actin antibody (QYA10733A) was available at Beijing Qualityard Biotechnology Co., Ltd. (Beijing, China).

### 4.2. Cell Culture and Treatment

KGN cells was supplied by Dr. Fenghua Liu (Guangdong Women and Children Hospital, Guangzhou, China). KGN cells were grown in DMEM/F-12 media with 8% FBS and maintained at 37 °C under 5% CO_2_. To establish a senescent model, KGN cells were exposed to different concentrations of D-gal (20, 40, 60, 80, 100 mg/mL) for 24 h [76]. To assess the toxic effects of HT, KGN cells were cultured with varying concentrations of HT (10, 20, 30, 40, 50 μM) for 24 h.

### 4.3. Cell Viabilities Assay

The viabilities of KGN cells were determined using the MTT method. KGN cells were plated in 96-well plates at a density of 1 × 10^5^ per well. After various treatments, 100 μL of MTT (0.5 mg/mL) was applied to each well, and the cells were grown at 37 °C for 4 h. After the media was removed, 150 µL of dimethyl sulfoxide was applied to each well and the plate was shaken for 8 min. MTT data were obtained by measuring the absorbance of each well at 490 nm via an enzyme labeller (BioTek, Winooski, VT, USA).

### 4.4. SA-β-Gal Assay

The SA-β-gal assay was conducted according to the manufacturer’s protocol. After the various treatments, the KGN cells were washed once with phosphate-buffered saline (PBS) and fixed with 1 mL of β-galactosidase staining fixative for 15 min at ambient temperature. After fixation, the cells were rinsed with PBS. Then, 1 mL of dyeing working solution (containing X-gal solution) was added to each well and the cells were incubated overnight at 37 °C in a thermostatic incubator. The cells were visualized with a normal light microscope. Blue SA-β-gal-positive cells were counted, and the proportion of blue SA-β-gal-positive cells was computed using Image J software Ver. 1.53q (Bethesda, MD, USA).

### 4.5. Measurement of ROS Production

Cells were plated on 12-well plates and stimulated with D-gal, either with or without HT, for 24 h. Following the various treatments, 10 μΜ of the DCFH-DA probe was applied to the KGN cells. The cells were incubated for 20 min and rinsed with PBS three times. The fluorescence images were obtained with a fluorescence microscope (Leica, Wetzlar, Germany). The average level of ROS was analyzed using Image J software.

### 4.6. Flow Cytometry Analysis

Following the various treatments, the KGN cells were gathered and centrifuged at 1000× *g* for 4 min. After discarding the supernatant, the cells were rinsed 3 times with pre-cooled PBS. The cells were suspended in 390 μL of Annexin V-FITC binding buffer containing 10 μL of Annexin V-FITC. Finally, 20 μL of propidium iodide was added. Flow cytometry (Beckman Coulter, Brea, CA, USA) was employed to measure the cell apoptosis. The apoptosis rate was equal to the rates of early and late apoptosis.

### 4.7. Animals and Treatments

Female C57BL/6 mice (aged 5–6 weeks) were acquired from the Guangdong Medical Laboratory Animal Centre (Foshan, China). The mice were reared in a controlled environment with a light-dark cycle of 12 h, respectively. The environmental conditions were carefully regulated, with a temperature of 22 °C and a moisture content ranging from 40 to 70%. All animal procedures were conducted according to the Guangdong Ocean University Laboratory Animal Care and Use Guidelines, and the experiments were authorized by the Guangdong Ocean University Animal Ethics Committee (Approval GDOU-LAE-2022-036).

After two weeks of acclimatization, mice with normal oestrous cycles were randomized into five groups (*n* = 15, per group): Control, D-gal, D-gal+ HT (50, 100, and 200 mg/kg/d, respectively) groups. Aside from the control group, mice were administered D-gal (400 mg/kg/d) via daily subcutaneous injections for a duration of 42 days [74]. Mice in the HT groups were administered the varying dosages of HT via the intragastric route from day 28 to day 56 for 28 consecutive days. Mice in the control group were orally administered the same volume of drinking water and subcutaneously injected with saline. The mouse body weights were recorded weekly at 10:00 a.m. The mice were anesthetized and sacrificed during the diestrus period after administration. Blood samples were taken via cardiac puncture, and the mouse ovaries were promptly removed and weighed. The right ovaries were refrigerated at −80 °C in order to facilitate biochemical investigation, while the left ovaries were immersed in a solution containing 4% paraformaldehyde to enable subsequent histological evaluation.

### 4.8. Oestrous Cycle Testing

From the third week of HT administration, the oestrous cycle was detected in the mice. Vaginal secretions were placed onto a slide, dried, and fixed with methanol. The vaginal secretions were dyed with H&E assay according to the manufacturer’s instructions, and visualized under a Leica dissecting microscope. The oestrous cycle was differentiated according to the following criteria: proestrus period, with large numbers of nucleated epithelial cells and few keratinized cells; estrus period, with non-nucleated keratinized cells or only a few epithelial cells; metestrus period, with leukocytes, keratinized cells, and nucleated epithelial cells; and diestrus period, a high leukocyte count and a low count of epithelial cells and mucous membranes.

### 4.9. Enzyme-Linked Immunosorbent Assay (ELISA)

Mouse blood samples were allowed to clot at room temperature for 2 h before centrifugation at 3000 rpm for 20 min to obtain serum. The collected serum was stored at −80 °C until analysis. The concentrations of FSH, E2, AMH, and P in the mouse serum were determined using ELISA kits as per the kit’s instructions.

### 4.10. Measurement of the SOD, MDA, and GSH Contents

After the various treatments, the KGN cells were sonicated with the addition of 1 mL of extraction solution. The supernatant was harvested by centrifuging the broken cells at 12,000 rpm for 10 min. The SOD and MDA contents in the supernatant were measured according to the kit guidelines. The ovaries were homogenized with PBS and the homogenates were centrifuged at 12,000 rpm for 15 min at 4 °C. The supernatants were preserved at −80 °C for subsequent biochemical analysis. GSH and MDA kits were used for measuring the GSH and MDA levels in the ovarian tissues, respectively.

### 4.11. H&E Staining and Follicle Number Counting

After sacrifice, ovary tissues were obtained and fixed with 4% paraformaldehyde overnight. The tissues were dehydrated in a graded ethanol series (75%, 85%, 90%, 95%, and absolute ethanol), followed by paraffin embedding according to standard histological procedures. Subsequently, the ovaries were sliced into 5-μm thick slides. The ovarian sections were subjected to histological staining using a H&E staining kit and were observed and photographed using a microscope. Serial sections were collected at regular intervals throughout the entire ovary, and follicles were counted only when the nucleus of the oocyte was clearly visible to avoid overestimation. Follicles were categorized and counted based on the following criteria: primordial follicle, a primary oocyte surrounding a layer of flattened follicle cells; primary oocyte, a central primary oocyte surrounding single-layer or multi-layer follicle cells; secondary follicles, oocyte encircled by more than one layer of cubic granule cells, but without a sinus cavity; antral follicle, a conspicuous cavity and an oocyte surrounding several layers of cuboidal granulosa cells; and atretic follicle, a follicle that does not undergo ovulation and instead undergoes a degenerative process. Based on the above descriptions, the quantification of primordial follicles, primary follicles, secondary follicles, antral follicles, and atretic follicles was calculated.

### 4.12. In Situ TUNEL Analysis

Ovarian granulosa cell apoptosis was detected in paraffin sections using a One-Step TUNEL Apoptosis Assay kit, in accordance with the kit instructions. For each group, five paraffin sections were selected at random. Finally, Image J software was employed to count the number of TUNEL-labelled granulosa cells in the ovarian sections.

### 4.13. Western Blotting

Ovarian tissues and KGN cells were lysed using a radio-immunoprecipitation solution. The protein lysates underwent centrifugation at 12,000 rpm for 15 min, resulting in the collection of the supernatant. Concentrations of protein samples were assayed by BCA kits.

Sodium dodecyl sulfate-polyacrylamide gel electrophoresis was carried out to separate the sample proteins. Target proteins were transferred to a polyvinylidene fluoride membrane after electrophoresis process was completed. Membranes were incubated in 5% skim milk at ambient temperature. After blocking for 2 h, the membranes were incubated with primary antibodies for 12 h at 4 °C. Subsequently, the membranes were cleaned six times with Tris-buffered saline solution before incubation for 2 h with the secondary antibody. The membranes were photographed using a chemiluminescence imaging system (Tanon-5200, Shanghai, China) following the application of ECL to the membranes. The intensity of the protein bands was standardized using β-actin as a reference.

### 4.14. Statistical Analysis

All data were analyzed using GraphPad Prism 8.0.2 software (San Diego, CA, USA) and expressed as Mean ± SEM. One-way analysis of variance (ANOVA) was performed where relevant. *p* < 0.05 was taken to indicate statistical significance.

## 5. Conclusions

**Figure 9:**
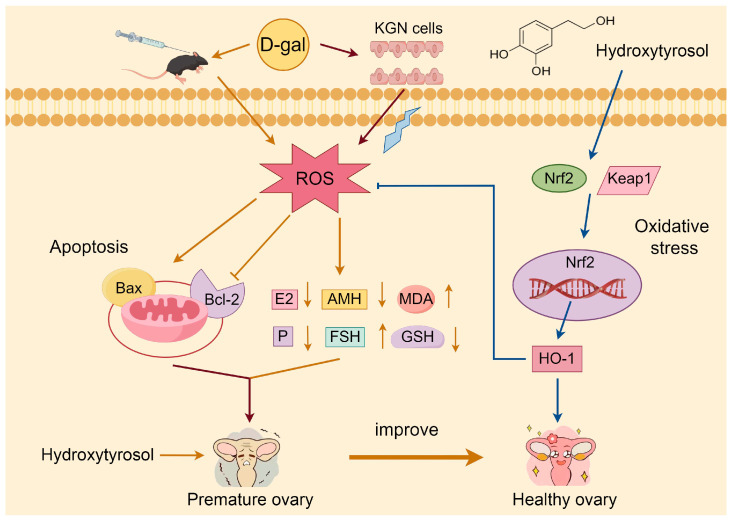
A schematic diagram showing the potential mechanism of HT in the D-gal-induced POI model (by www.figdraw.com [accessed 18 May 2026]). HT treatment may alleviate oxidative stress and granulosa cell apoptosis in D-gal-induced POI, potentially through modulation of the Nrf2/HO-1 signalling pathway, thereby contributing to the restoration of ovarian function.

In conclusion, hydroxytyrosol, the primary phenolic compound derived from olive oil, alleviated D-galactose-induced premature ovarian insufficiency-like changes in vitro and in vivo. Hydroxytyrosol treatment improved oxidative stress status, reduced granulosa cell apoptosis, restored ovarian function-related parameters, and was associated with modulation of the Nrf2/HO-1 signalling pathway (Figure 9). These findings provide preliminary evidence supporting the potential protective effects of HT in POI models. However, the current study does not establish a direct causal relationship between Nrf2/HO-1 activation and the observed biological effects, and additional mechanistic studies are needed to further validate the underlying pathways and therapeutic potential of HT.

## Data Availability

The data presented in this study are available on request from the corresponding author.

## Abbreviations

The following abbreviations are used in this manuscript:

AGEs	advanced glycation end products
AMH	anti-Müllerian hormone
Bax	Bcl2 associated X protein
Bcl-2	B-cell lymphoma 2
D-gal	D-galactose
E2	oestradiol
EVOO	extra virgin olive oil
FSH	follicle-stimulating hormone
GCs	granulosa cells
GSH	glutathione
H&E	hematoxylin and eosin
HO-1	heme oxygenase-1
HPG	hypothalamic-pituitary-gonad
HPO	hypothalamic-pituitary-ovarian
HRT	hormone replacement therapy
HT	hydroxytyrosol
KGN cells	human ovarian granulosa-like tumour cell line
Keap1	Kelch-like ECH-associated protein 1
MD	Mediterranean diet
MDA	malondialdehyde
MTT	3-(4,5-dimethylthiazol-2-yl)-2,5-diphenyltetrazolium bromide
Nrf2	nuclear factor erythroid 2-related factor 2
P	progesterone
PBS	phosphate-buffered saline
POI	premature ovarian insufficiency
ROS	reactive oxygen species
SA-β-gal	senescence-associated β-galactosidase
SOD	superoxide dismutase
